# Antioxidant-rich Clove Extract, A Strong Antimicrobial Agent against Urinary Tract Infections-causing Bacteria *in vitro*

**DOI:** 10.21315/tlsr2021.32.2.4

**Published:** 2021-06-29

**Authors:** Vanessa Lee Rosarior, Pei Shan Lim, Wei Kit Wong, Chen Son Yue, Hok Chai Yam, Sheri-Ann Tan

**Affiliations:** 1 Department of Bioscience, Faculty of Applied Sciences, Tunku Abdul Rahman University College, Jalan Genting Kelang, 53300 Setapak, Kuala Lumpur, Malaysia; 2 Department of Physical Science, Tunku Abdul Rahman University College, Jalan Genting Kelang, 53300 Setapak, Kuala Lumpur, Malaysia; 3 Faculty of Applied Sciences, UCSI University Kuala Lumpur Campus (South Wing), No.1, Jalan Menara Gading, UCSI Heights, Taman Connaught, 56000 Cheras, Kuala Lumpur, Malaysia

**Keywords:** Antimicrobial, Antioxidant, Clove, Essential Oil, Phenolic Compounds, Antimikrob, Antioksidan, Bunga Cengkih, Minyak Pati, Kompaun Fenolik

## Abstract

Clove (*Syzygium aromaticum*) is an exotic culinary spice that has been used for centuries due to its known antimicrobial and antioxidant properties. The main aim of this study is to compare the antimicrobial activity and antioxidant capacity of clove ethanolic extract (CEE) and commercial clove essential oil (CEO) at a standardised eugenol content. Disk diffusion assay showed that CEE (2000 μg) was able to exhibit broad-spectrum inhibition against both Gram negative and Gram positive Urinary Tract Infections (UTIs)-causing pathogens: *Proteus mirabilis* (19.7 ± 0.6 mm) > *Staphylococcus epidermidis* (18 mm) > *Staphylococcus aureus* (14.7 ± 0.6 mm) > *Escherichia coli* (12.7 ± 0.6 mm) > *Klebsiella pneumoniae* (12.3 ± 0.6 mm) (according to the size of inhibition zone). Interestingly, the comparison between CEE and commercial CEO revealed that the former demonstrated stronger antimicrobial and antioxidative properties at similar eugenol concentration. The EC_50_ of DPPH (1,1-diphenyl-2-picrylhydrazyl), ABTS (2,2′-azino-bis(3-ethylbenzothiazoline-6-sulfonic acid) and reducing power assay for CEE were determined as 0.037 mg/mL, 0.68 mg/mL and 0.44 mg/mL, respectively. Besides eugenol, High Performance Liquid Chromatography (HPLC) analyses identified the presence of kaempferol, gallic acid and catechin in CEE. As a conclusion, we concluded that there was a possible synergistic effect between eugenol and the others active compounds especially kaempferol which led to the observed bioactivities in CEE.

## Abstract (fr)

Bunga cengkih (*Syzygium aromaticum*) merupakan salah satu rempah masakan eksotik yang telah digunakan berabad-abad untuk kegunaan antimikrob dan antioksidan. Matlamat utama kajian ini adalah untuk membandingkan aktiviti antimikrob dan kapasiti antioksidan di antara ekstrak etanol bunga cengkih (CEE) dan minyak pati bunga cengkih komersial (CEO) dengan kandungan eugenol yang sama. Pencerakinan resapan agar menunjukkan CEE mempunyai perencatan spektrum yang luas terhadap bakteria Gram negatif dan Gram positif, patogen penyebab jangkitan saluran kencing: *Proteus mirabilis* (19.7 ± 0.6 mm) > *Staphylococcus epidermidis* (18 mm) > *Staphylococcus aureus* (14.7 ± 0.6 mm) > *Escherichia coli* (12.7 ± 0.6 mm) > *Klebsiella pneumoniae* (12.3 ± 0.6 mm) (menurut saiz zon perencatan). Yang menarik, perbandingan CEE dan CEO mendedahkan bahawa CEE menunjukkan aktiviti antibakteria yang kuat. Hapus-sisa radikal bebas DPPH dan ABTS serta aktiviti kuasa redaksi untuk rempah ini telah dibandingkan dengan CEO. Keputusan menunjukkan aktiviti antioksidan dalam CEE adalah lebih kuat. EC_50_ DPPH, ABTS dan pencerakinan kuasa redaksi untuk CEE masing-masing telah ditentukan sebagai 0.037 mg/mL, 0.68 mg/mL and 0.44 mg/mL. Kompaun aktif (eugenol dan lainlain kompaun fenolik) merupakan kompaun yang terkandung dalam CEE. Analisis HPLC mengkuantitikan kehadiran kaempferol, asid galik dan katechin. Kesimpulannya, kita menjangkakan kemungkinan terdapat kesan sinergi di antara eugenol dengan kompaun fenolik lain terutamanya kaempferol yang berupaya meningkatkan aktiviti CEE berbanding dengan CEO.

**Highlights**

- Clove ethanolic extract (CEE) exhibits antimicrobial activities towards Gram negative and Gram positive UTI-causing bacteria.
- CEE is more effective than commercial clove essential oil in terms of antimicrobial and antioxidant activities.
- Synergistic effect between eugenol, kaempferol, gallic acid and catechin present in CEE could possibly exert the observed bioactivities.

## INTRODUCTION

Cloves (*Syzygium aromaticum*) originate from Maluku Island in Indonesia but nowadays they are widely distributed in several regions around the world. Clove has been utilised in traditional Chinese medicine as a remedy for stomach ailments such as inflammation and diarrhoea (Kamatou *et al*. 2012). Clove oil containing the major component eugenol has been reported to demonstrate many therapeutic properties which include antifungal, antibacterial, antioxidant, hepatoprotective and even anticancer effects (Mittal *et al*. 2014; Kamatou *et al*. 2012; Souza *et al*. 2013; Goñi *et al*. 2009). The pure compound itself, eugenol has also been mentioned to possess these beneficial activities (Ishaq *et al*. 2019; Pavesi *et al*. 2018).

Clove in the form of essential oil and even eugenol alone are found to be free from any toxic effects. Various acute and chronic toxicity studies of clove oil conclude no adverse effects on albino rats (Shalaby *et al*. 2011). Eugenol is in fact shown to be rapidly absorbed, metabolised in the liver and eliminated within 24 h when consumed orally (Cortés-Rojas *et al*. 2014). A recent toxicity assessment of the water-soluble polyphenol rich extract powder of dried clove buds (known as ‘Clovinol’), containing 41.2% gallic acid equivalent of polyphenols indicates that this preparation is safe in rats (Vijayasteltara *et al*. 2016). In short, the clove preparations regardless in the form of an extract or just its essential oil are free from toxicity.

Urinary tract infections (UTIs) are commonly caused by members of the family Enterobacteriaceae such as *Escherichia coli*, *Proteus mirabilis*, *Klebsiella pneumoniae* as they are the most frequently isolated bacteria in the clinical laboratory (Zhanel *et al*. 2000). Uropathogenic *Escherichia coli* (UPEC) is responsible for more than 80% of community acquired UTIs (Barber *et al*. 2013). This infection is widespread in developing countries like Malaysia and also occurs in developed nations like the United States (Schulz *et al*. 2016). About seven million cases of UTIs are diagnosed annually in the United States, where females are found to be most likely infected. This number also generally includes patients who acquire nosocomial UTIs (Medina & Castillo-Pino 2019).

Common antibiotics used in preventing the widespread of the infections are trimethoprim-sulfamethoxazole, nitrofurantoin monohydrate and fosfomycin trometamol. Intravenous administration is performed when an infection is too severe (Barber *et al*. 2013; Zhanel *et al*. 2000). Nevertheless, when these bacteria are exposed to antibiotics over a long period of time, they develop multiple ways of becoming resistant to these drugs. Although the resistance to antibiotics by bacteria is a natural adaptation phenomenon, the emergence of multidrug-resistant (MDR) bacteria is steadily increasing and threatening the world population (Voukeng *et al*. 2017). This is mainly due to the misuse of antibiotics, inadequate dosage, low quality of antibiotics and poor patient compliance (Bisi-Johnson *et al*. 2017). As a consequence, many UTIs causing bacterial strains to have evolved to MDR bacteria such as *Klebsiella pneumoniae carbapenemase* (KPC) producing bacteria and methicillin resistant *Staphylococcus aureus* (MRSA). UTI isolates from Canada and the United States are also discovered to be resistant towards ampicillin and trimethoprim-sulfamethoxazole (Zhanel *et al*. 2000). To overcome the phenomenon of increasing bacterial resistance to existing drug, one of the possible ways is the search for new antimicrobial agents, especially from the natural sources (Imran *et al*. 2017). In view of that, natural spices could be used to overcome the limitations of modern antibiotics. Unlike antibiotics, the antimicrobial agents from natural origins have less or no side effects and thus they have a good therapeutic potential as natural alternative to treat UTIs.

Our preliminary study proves that clove ethanolic extract (CEE) is an effective antimicrobial agent against Gram-positive (*Staphylococcus epidermidis*, *Staphylococcus aureus*) and Gram-negative (*Proteus mirabilis*, *Escherichia coli*, *Klebsiella pneumoniae*) UTIs-causing pathogens when compared to clove essential oil (CEO). This extract also exhibits strong antioxidant activities. The active compounds elucidated from the CEE are eugenol and phenolic compounds such as kaempferol, gallic acid and catechin.

## MATERIALS AND METHODS

### Materials

*Syzygium aromaticum* (cloves) were purchased from the local wholesale store, New Group Medicine Sdn. Bhd. located in Cheras, Kuala Lumpur, which imported this spice from Gong-sai, China. Clove essential oil was purchased from NOW Company, United States.

### Bacteria Strains and Reagents

Six bacteria strains were used to examine the antibacterial properties of the test samples; four Gram-negative [*Escherichia coli* (ATCC 25922), *Klebsiella pneumoniae* (ATCC 13883), *Proteus mirabilis* (ATCC 43071), *Pseudomonas aeruginosa* (ATCC 10145)] and two Gram-positive [*Staphylococcus aureus* (ATCC 25923) and *Staphylococcus epidermidis* (ATCC 12228)]. All bacteria strains were obtained from UCSI University, Kuala Lumpur Campus (South Wing). All chemicals and solvents used were of analytical or HPLC grade. Gallic acid was purchased from Merck (Germany). (+)-Catechin were purchased from Sigma-Aldrich (USA) and kaempferol were obtained from EMD Millipore (USA). HPLC grade methanol was obtained from Fluka (USA) and ultra-pure water was produced from the Mili-Q water purification system (18 m′Ω cm, Merck Millipore, USA).

### Phytochemicals Extraction

Clove spices were washed with sterile water and dried at 40°C to the constant of mass. Then, the spice was ground with a mixer into fine powder and stored in an airtight container at 4°C (Rakshit & Ramalingam 2010). Maceration was performed using 80% ethanol at a ratio of 1:5 (w:v) for 24 h with occasional shaking. The filtrate was then concentrated using a rotary evaporator (Buchi R-100) before proceeding with freeze drying (Coolsafe 110–4, Labogene). The extract obtained was then stored in the dark at 4°C. Prior to antimicrobial and antioxidant assays, the extract was first dissolved in 10% of dimethylsulfoxide (DMSO) into the concentrations required.

### Determination of Antibacterial Activity of CEE

#### Disk diffusion assay

An amount of 150 μL of bacterial culture was uniformly spread on the surface of Mueller-Hinton agar according to Kirby Bauer method as mentioned in Clinical and Laboratory Standards Institute (CLSI) (2020). The plates were dried for 15 min before placing 7 mm paper disc that had been impregnated with 200 μg, 1000 μg and 2000 μg of CEE. Chloramphenicol (30 μg) antibiotic discs (Oxoid) were used as positive controls and 10% dimethylsulfoxide (DMSO) was used as a negative control. The plates were then incubated at 37°C ± 1°C for 24 h under aerobic condition and zone of inhibition was measured to the nearest millimeters (mm) (Mendez-Vilas 2011). All disk diffusion assays for respective bacteria were performed in triplicates and the antibacterial activity was expressed as the mean of clear inhibition zones diameters (mm) produced by the test substances.

#### Microwell dilution assay

The bacterial suspension was adjusted to give a turbidity of 0.5 McFarland standards which contained approximately 1.5 × 10^8^ CFU/mL as recommended by CLSI guidelines. The concentrations prepared for CEE and CEO were as such: 10 mg/mL, 5 mg/mL, 2.5 mg/mL, 1.25 mg/mL, 0.625 mg/mL, 0.313 mg/mL, 0.156 mg/mL and 0.078 mg/mL. Sample was added into each respective well followed by the bacterial inoculum. Turbidity was observed after a 24-hour incubation (Balouiri *et al*. 2016).

### Determination of Antioxidative Activities of Clove Ethanolic Extract (CEE) and Clove Essential Oil (CEO)

#### DPPH and ABTS radical scavenging assays

CEE and CEO samples were standardised at different eugenol concentrations based on the GC-MS peak area for this compound (0.09%, 0.12% and 0.34%). For DPPH radical scavenging assay, a total of 1 mL sample was added into 2 mL of 0.15 mM of DPPH according to Lim and Murtijaya (2007). The mixture was incubated for 30 min before measuring the absorbance at 517 nm. The procedure for ABTS assay was based on El-Maati *et al*. (2016) with minor modifications. The photometric assay was conducted by mixing 1 mL of sample with 2 mL of ABTS+ radical solution. The measurement was taken after a 7-minute incubation period at absorbance 734 nm. The radical scavenging activities for both assays were calculated based on the equation below:

$$\text{Scavenging activity }(\%)=[({\text{A}}_{\text{control}}-{\text{A}}_{\text{sample}})/{\text{A}}_{\text{control}}]\times 100\%$$

Where A_control_ = Absorbance reading of control group and A_sample_ = Absorbance of sample group.

#### Reducing power assay

In reducing power assay, the samples were first mixed with phosphate buffer to a volume of 2.5 mL and then with potassium ferricyanide and ferric chloride. Increase in absorbance indicated an increase in antioxidant activity (Jayanthi & Lalitha 2011).

### Phytochemical Analyses of the CEE

#### Total phenolic (TPC) and flavonoid contents (TFC)

TPC and TFC were obtained based on Folin-Ciocalteau and aluminium chloride colorimetric procedures, respectively (Biju *et al*. 2013) using 2.5 mg/mL of CEE. Results were expressed in Gallic Acid Equivalents (GAE) for TPC and Catechin Equivalents (CE) for flavonoids content.

#### Bioactive phytochemical screening

CEE was analysed for the presence of saponins, alkaloids, fixed oil, tannins and phenolic compounds, terpenoids, cardiac glycosides and flavonoids. The tests performed were: Foam test (Mandal *et al*. 2013); Wayner’s test (Khanam *et al*. 2015) and Mayer’s test (Jeyaseelan & Jashothan 2012); Stain test (Raaman 2006); Ferric chloride test (Mandal *et al*. 2013), Salkowski’s test (Jeyaseelan & Jashothan 2012); Keller-Kiliani’s test (Ismail *et al*. 2016); Shinoda test (Gangwar *et al*. 2014).

### GC-MS Analysis

The compounds of CEE and CEO were analysed by GC-MS performed on Agilent Technologies 7890A GC system (Agilent Technologies, California, US). Filtered samples were transferred into GC-MS vials prior to analysis. The GC-MS was equipped with a flame ionization detector (FID) and a fused silica capillary column HP5MS (5% phenylmethylpolysiloxane, 30 m × 0.25 mm ID, 0.25 μm film thickness). In this case, the carrier gas used was helium, at a flow rate of 1 mL/min. Injection port temperature was 250°C. Column temperature was programmed as: 50°C (2 min) isotherm, increased to 250°C at a rate of 10°C/min and held at 280°C for 15 min. The mass spectrometer was operated in the electron impact ionization mode. The chromatographic spectrum was analysed based on NIST08.L database of the MSDChem workstation (Zhang *et al*. 2015). Qualitative standardisations of CEE and commercial CEO were performed using this technique. After the peak area of eugenol at specific concentration was obtained, a standard graph of concentrations of CEO against eugenol peak area was determined.

### HPLC Analysis

The identification analyses of phenolic compounds were conducted using a HPLC unit (Agilent Technologies, California, US) that consisted of a Agilent 1260 Infinity Quaternary Pump, a 1260 Infinity Diode-Array Detector, a Agilent 1260 Infinity Standard Autosampler Injector with a loop of 20 μL and a reversed phase Eclipse Plus C18 column (250 mm × 4.6 mm × 5 μm). An isocratic elution consisting of ultrapure water with 0.1% orthophosphoric acid and HPLC grade methanol in the ratio of 80:20 over 30 min was used. The flow rate, wavelength and column temperature were set at 1.0 mL min^−1^, 210 nm and 30°C, respectively. This method was modified from Wang *et al*. (2000).

### Statistical Analysis

Experiments were triplicated and the results were expressed as mean ± standard error. Statistical analyses of data were as followed: prior to analysis, the data were tested for homogeneity of variances by the test of Levene; for multiple comparisons, one-way analysis of variance (ANOVA) was performed. The level of significance was *p* < 0.05. SPSS version 21.0 was used. EC_50_ value was determined using GraphPad Prism 7.0 (GraphPad Software, California, USA).

## RESULTS

### Determination of Antibacterial Activity of CEE

**Figure 1:**
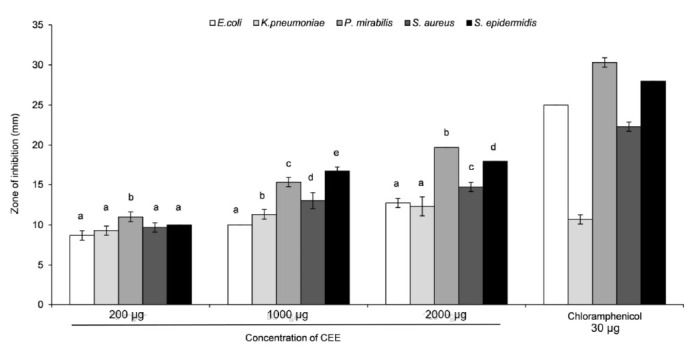
Zone of inhibition test of various concentrations of CEE against different UTIscausing pathogens. Chloramphenicol was used as a positive control and 10% DMSO as negative control. Data is expressed as mean ± SEM (*n* = 3). The data that do not share the same letter are significantly different with *p-*value < 0.05 within classes of treatment at similar concentration.

CEE was evaluated for their antimicrobial properties using the Kirby-Bauer disc diffusion assay on six UTIs causing bacteria. Based on the result illustrated in Fig. 1, CEE exhibited broad spectrum inhibition of five UTIs causing pathogens of both Gram-positive and Gram-negative strains. The pathogen which was most susceptible towards CEE was *Proteus mirabilis*, a Gram-negative bacterium, with a zone of inhibition of 19.7 mm ± 0.6 followed by *Staphylococcus epidermidis* (18.0 mm ± 0.6), *Staphylococcus aureus* (14.7 mm ± 0.6), *Escherichia coli* (12.7 mm ± 0.6) and *Klebsiella pneumoniae* (12.3 mm ± 0.6). However, no zone of inhibition was observed on *Pseudomonas aeruginosa* disc diffusion plate.

Microwell dilution assay was carried out on two of the most susceptible pathogens towards CEE to determine the MIC. The MIC obtained for both *Proteus mirabilis* [Gram (-)] and *Staphylococcus epidermidis* [Gram (+)] were 0.313 mg/mL. CEE at the concentration of 0.313 mg/mL was analysed using GC-MS to obtain its eugenol peak area. The identified eugenol peak area in CEE sample at that concentration was 0.34%.

A qualitative standardisation was then carried out between CEE and a commercial CEO based on the eugenol content using GC-MS. The peak areas of eugenol present in CEO at the concentrations 0.05 mg/mL, 0.10 mg/mL, 0.15 mg/mL, 0.20 mg/mL and 0.25 mg/mL were obtained. A standard curve graph was constructed using the respective CEO concentrations against its peak area (Supp. Mat. 1). According to the standard curve, the eugenol peak area obtained for CEE at the concentration of 0.313 mg/mL corresponded to 0.15 mg/mL concentration of CEO. Thus, the percentage of eugenol in the CEO was higher as compared to the amount presence in CEE at similar concentration. Microwell dilution assay was conducted on CEO to obtain its MIC value. It was found that MIC of CEO was 0.313 mg/mL for both *Proteus mirabilis* and *Staphylococcus epidermidis*. This clearly indicated that eugenol was not the only compound responsible for the observed antimicrobial effect in CEE. If eugenol was the compound exerting antimicrobial effect, thus the MIC for CEO would theoretically be 0.15 mg/mL.

### Determination of Antioxidative Activities of Clove Ethanolic Extract (CEE) and Clove Essential Oil (CEO)

**Figure 2:**
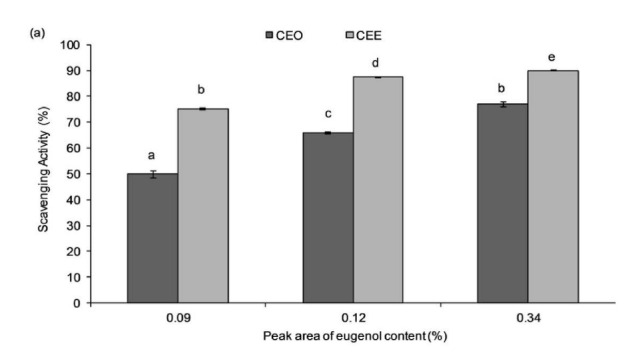

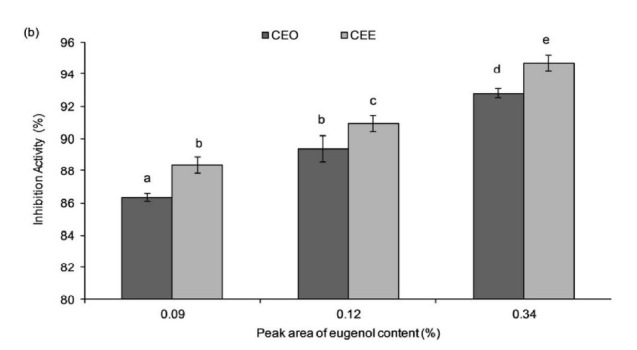

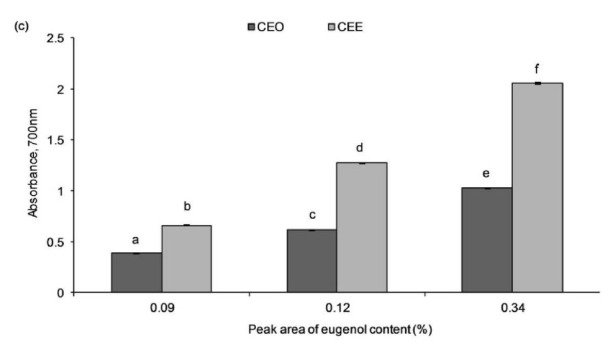
(a) DPPH and (b) ABTS radical scavenging activities as well as (c) reducing power capacity were evaluated on both CEE and CEO at standardised eugenol concentration. Data is expressed as mean ± SD (*n* = 3). The data that do not share the same letter are significantly different with *p*-value < 0.05.

Chemical-based antioxidant assays testing DPPH and ABTS scavenging activities as well as reducing power capacity were performed on CEE and CEO at standardised eugenol concentrations based on GC-MS peak area for this compound (0.09%, 0.12% and 0.34%). Results from the antioxidative assays demonstrated a dosage-dependent response in which the increase of the extract concentrations resulted in higher antioxidant effects of CEE and CEO (Fig. 2). CEE exhibited stronger radical scavenging ability and better reducing potential as compared to CEO at similar eugenol concentration. The EC_50_ of DPPH, ABTS and reducing power assay for CEE were determined as 0.037 mg/mL, 0.68 mg/mL and 0.44 mg/mL, respectively. CEE was further tested for its phytochemicals content.

### Phytochemical Analyses of CEE

**Figure 3:**
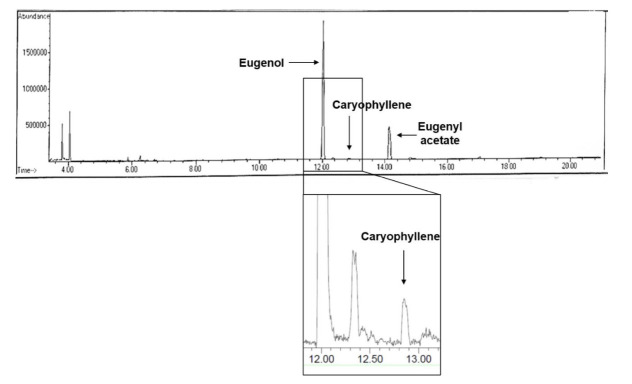
The GC-MS total current ion chromatogram of compounds from CEE. The components identified were eugenol, caryophyllene and eugenyl acetate. All the compounds were elucidated based on the NIST 08 library.

As deduced from the phytochemical screening result, CEE contained fixed oil, tannins, phenolic compounds, terpenoids, cardiac glycosides but saponin was not detected. The total phenolic content of CEE was determined to be 250.93 ± 1.33 mg Gallic Acid Equivalent (GAE)/g extract while its total flavonoid content was 57.34 ± 1.33 mg Catechin Equivalent (CE)/g extract. Further chemical analysis of this spice by GC-MS led to the identification of major components in CEE; eugenol, eugenyl acetate and caryophyllene (Fig. 3).

Phenolics compounds presence in CEE was evaluated using HPLC along with chemical standards. The peak identification was based on the comparison of the retention time between the CEE sample and standard phenolic compounds (Supp. Mat. 2). Quantitation of phenolic compounds such as kaempferol, catechin and gallic acid was performed on crude CEE shown in Table 1.

## DISCUSSION

Phytochemical extraction for Clove spice was carried out using aqueous-ethanolic maceration as several studies revealed that 80% ethanol was able to extract most of the bioactive phytochemical compounds especially flavonoids effectively (Valle *et al*. 2016; Wang *et al*. 2011). Previous research compared different solvent extraction on spices and reported that 80% ethanolic extraction method showed highest inhibition towards both Gram-negative and Gram-positive bacteria (El-Maati *et al*. 2016).

Disc diffusion method was selected to determine the antimicrobial ability of CEE. According to Clinical and Laboratory Standards Institute (CLSI) handbook 2020, the diameter of the inhibition zone for the tested bacterial strains categorised as susceptible towards chloramphenicol (30 μg) was 18 mm and above, intermediate was between 13 mm to 17 mm, whereas 12 mm and below was considered as resistance against chloramphenicol. Based on our results, *Proteus mirabilis* and *Staphylococcus epidermidis* were both categorised as susceptible towards CEE at 2000 μg, since the zones of inhibition were 19.7 ± 0.6 mm and 18 ± 0.0 mm, respectively. On the other hand, *Staphylococcus aureus* and *Escherichia coli* were categorised as intermediate breakpoint, and *Klebsiella pneumoniae* and *Pseudomonas aeruginosa* were categorised under resistance breakpoint at the same extract amount. Similar work was done by Nascimento *et al*. (2000) using Clove ethanolic extract prepared at solvent ratio 1:1 (w/v). Their extract demonstrated antimicrobial activities against *Staphylococcus aureus* ATCC 6538, *Pseudomonas aeruginosa* ATCC 15442 and *Klebsiella pneumoniae*. Bioactive compounds reported in the extract were eugenol, flavonoids and tannins. This bioactivity could be attributed to the major clove compound, eugenol. The hydrophobicity of eugenol could break down the cellular lipid and damage the bacterial cell wall, resulting in cell lysis and leakage of intracellular fluids (Ishaq *et al*. 2019; Pavesi *et al*. 2018).

Determination of the minimum inhibitory concentrations (MIC) of CEE was next performed on the UTIs-causing pathogens which were most susceptible to the extract; *Proteus mirabilis* and *Staphylococcus epidermidis* using broth-dilution method (Balouiri *et al*. 2015). By comparing the wells of each bacterium, it was concluded that CEE possessed similar MIC for *Proteus mirabilis* and *Staphylococcus epidermidis* which was at 0.313 mg/mL.

Higher amount of eugenol was presence in CEO as compared to CEE at similar concentration. Theoretically, if eugenol was the only compound responsible for the antimicrobial activity, the deduced MIC value for CEO would be 0.15 mg/mL. Nevertheless, the MIC for CEO was found to be 0.313 mg/mL. In view of that, we postulated that other bioactive molecules specifically phenolic compounds were working synergistically with eugenol to cause the observed antimicrobial properties of CEE. A number of antimicrobial works had been conducted on clove oil. Previous study by Goñi *et al*. (2009) demonstrated that a commercial clove oil rich in eugenol at 10 mg was found active against foodborne Gram-positive (*Staphylococcus aureus*, *Bacillus cereus*, *Enterococcus faecalis* and *Listeria monocytogenes*) and Gram-negative bacteria (*Escherichia coli*, *Yersinia enterocolitica* and *Salmonella choleraesuis*). Related research by Souza *et al*. (2013) investigated the inhibitory activity of clove essential oil purchased from Ferquima (Brazil) against two of the commonly found fungi in bread, *Penicillium commune* and *Eurotium amstelodami*. The alcoholic solution of this essential oil at 16 g/100 g was found to exhibit complete inhibition on both fungi tested. Since our findings suggested that CEE possessed better antimicrobial activity as compared to CEO at similar eugenol content, antioxidant assays were also performed to compare the activities of these two samples. Urinary tract infection had been reported to cause oxidative stress by depleting the urinary antioxidant enzymes (Kurutas *et al*. 2005). If CEE could confer strong antioxidative effects besides exerting antimicrobial properties towards UTIs-causing pathogens, it certainly possessed the potential to be developed into an effective treatment against this infection.

When the amount of eugenol content was standardised in CEE and CEO, it was found that CEE again possessed better antioxidant activity than CEO based on their DPPH and ABTS radical scavenging properties as well as reducing power effects. The evidence presented thus far supported the idea that other compounds could also be responsible for both observed phenomena. Therefore, we postulated a synergistic effect occurring between eugenol and other phenolic compounds found abundantly in CEE. Eugenol had already been reported to exhibit a positive synergistic antimicrobial effect on different bacterial strains when coupled with different types of antibiotics such as fluconazole, tetracycline and colistin (Ahmad *et al*. 2010; Miladi *et al*. 2017; Wang *et al*. 2018).

A correlation study between isolated plant components and inhibition of microorganism confirmed that phenolic compounds possessed strong antibacterial activities (Dorman & Deans 2000). Thus, high amount of phenolic content detected in CEE might be one of the major factors contributing to the strong antimicrobial properties in both Gram-positive and Gram-negative UTIs-causing bacteria tested. In our work we had identified the presence of other phenolics in CEE besides eugenol. Kaempferol (5.839 mg/g CEE) was detected in high concentration in CEE as compared to other phenolic compounds. Both catechin and gallic acid were presented in a smaller amount; 0.0184 mg/g CEE and 0.0169 mg/g CEE, respectively. Many profound pharmacological research had been conducted on kaempferol along with its glycosides and they showed antioxidant, anti-inflammatory, antimicrobial, anticancer as well as analgesic properties (Baliga *et al*. 2013). Several pathogens such as *Enterococcus faecalis* (ATCC 29212), *Staphylococcus aureus* (ATCC 29213), *Escherichia coli* (ATCC 27853) and *Pseudomonas aeruginosa* (ATCC 25922) were found to be susceptible with MIC values ranging between 16 μg/mL–63 μg/mL when tested with isolated kaempferol from *Dodonaea viscosa* leaf extracts (Teffo *et al*. 2010). It was described that kaempferol inhibited *Staphylococcus aureus* by reducing the adhesion of bacteria to fibrinogen thus hindering the formation of biofilms (Ming *et al*. 2017).

We hypothesised synergism between eugenol and kaempferol contributed to the high antimicrobial and antioxidant effects of CEE. In fact, Burt (2004) had reported that minor components like kaempferol were crucial factors in conferring antimicrobial activities as they may possess synergistic effect or potentiating influence with other phytochemicals. A cocktail of substances indeed will have a stronger effect as compared to its individual counterpart.

## CONCLUSION

Clove ethanolic extract (CEE) indeed possessed effective *in vitro* antimicrobial properties against Urinary Tract Infections (UTIs)-causing bacteria especially *Proteus mirabilis* and *Staphylococcus epidermis*. At similar eugenol concentration, CEE even performed better than the commercial clove essential oil as a broad-spectrum antimicrobial and antioxidant agent. In view of that, there could be a possible synergism between eugenol and kaempferol present in CEE contributing to the observed activities. As such, further study on the mechanism of action between these two compounds is required to validate their synergistic action. Furthermore, utilising an *in vivo* model system for UTI in future work would significantly enhance research findings to support these bioactives as treatment for this ailment.

## APPENDICES

Supplementary Material 1 The standard curve of eugenol peak area (%) in clove essential oil (CEO) with R^2^ of 0.988.

Supplementary Material 2 HPLC-DAD chromatograms of (a) standard solution of gallic acid (100 μg/mL) (b) standard solution of catechin (100 μg/mL) (c) standard solution of kaempferol (100 μg/mL) and (d) clove ethanolic extract (CEE).

## Figures and Tables

**Table 1:** Amount (mg) of phenolic compounds per gram of CEE determined by HPLC-DAD analysis.

Phenolic compounds	Concentration of phenolic compounds
Kaempferol	5.839 mg/g CEE
Catechin	0.0184 mg/g CEE
Gallic acid	0.0169 mg/g CEE
