# Supplementary figures and images for: Antioxidant-rich Clove Extract, A Strong Antimicrobial Agent against Urinary Tract Infections-causing Bacteria in vitro
Source: Trop Life Sci Res. 2021 Jun 29;32(2):45–63. doi: 10.21315/tlsr2021.32.2.4 (PMC8300942; doi:10.21315/tlsr2021.32.2.4)

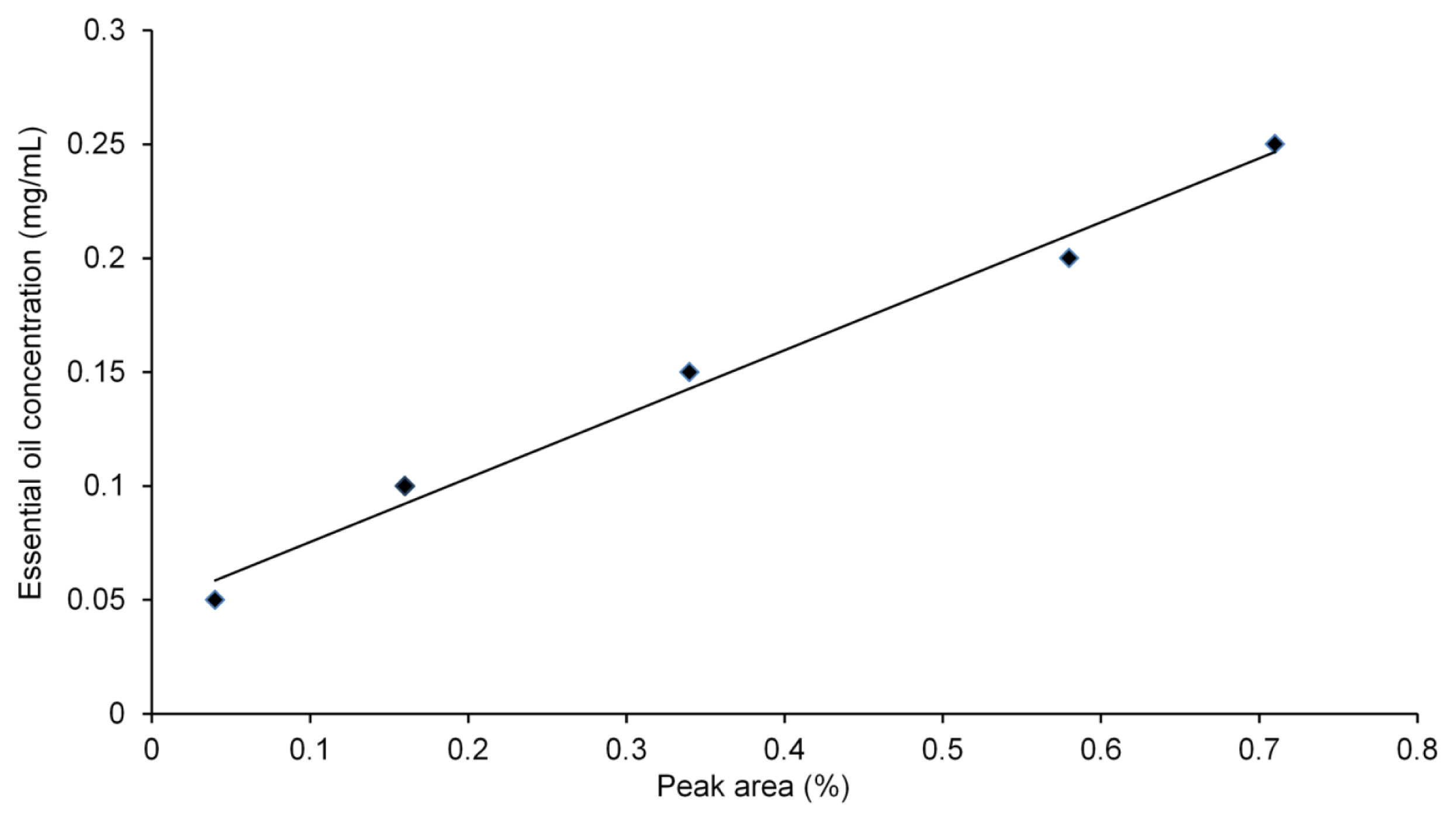

Supplement: Supplementary Material 1 — The standard curve of eugenol peak area (%) in clove essential oil (CEO) with R2 of 0.988. [file TLSR-32-2-45-s001.tif]

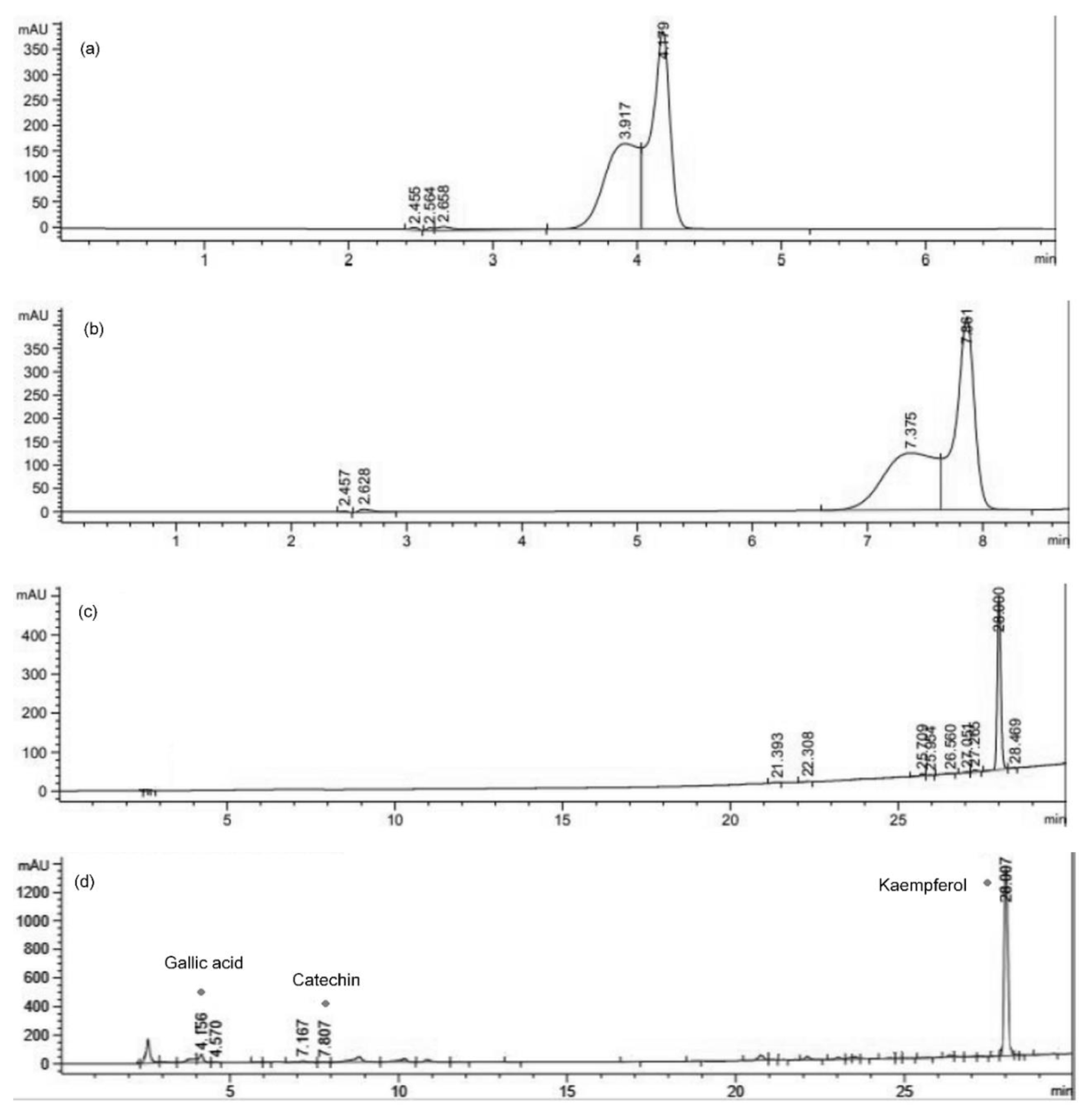

Supplement: Supplementary Material 2 — HPLC-DAD chromatograms of (a) standard solution of gallic acid (100 μg/mL) (b) standard solution of catechin (100 μg/mL) (c) standard solution of kaempferol (100 μg/mL) and (d) clove ethanolic extract (CEE). [file TLSR-32-2-45-s002.tif]
